# Supplementary material for: The Methylene Spacer Matters: The Structural and Luminescent Effects of Positional Isomerism of n-Methylpyridyltriazole Carboxylate Semi-Rigid Ligands in the Structure of Zn(II) Based Coordination Polymers
Source: Polymers (Basel). 2023 Feb 10;15(4):888. doi: 10.3390/polym15040888 (PMC9961080; doi:10.3390/polym15040888)
Supplement: Supplementary file 1 [file polymers-15-00888-s001.zip › polymers-2158679-supplementary-revised.pdf]

ELECTRONIC SUPPLEMENTARY INFORMATION

The methylene spacer matters: the structural and luminescent effects of positional isomerism of n-methylpyridyltriazole car-boxylate semi-rigid ligands in the structure of Zn(II) based co-ordination polymers

Pilar Narea[1], Benjamín Hernández[1], Jonathan Cisterna[2], Alejandro Cárdenas[3], Pilar Amo-Ochoa[4,5], Félix Zamora[4,5,6], Gerzon E. Delgado[1,7], Jaime Llanos[2] and Iván Brito[1]*

[1]Departamento de Química, Facultad de Ciencias Básicas, Universidad de Antofagasta, Casilla 170, Antofagasta 1240000, Chile
[2] Departamento de Química, Facultad de Ciencias, Universidad de Católica del Norte, Sede Casa Central, Av. Angamos 0610, Antofagasta, Chile
[3]Departamento de Física, Facultad de Ciencias Básicas, Universidad de Antofagasta, Casilla 170, Antofagasta 1240000, Chile
[4]Departamento de Química Inorgánica, Universidad Autónoma de Madrid, 28049 Madrid, Spain
[5]Institute for Advanced Research Chemistry (IAdChem), Universidad Autónoma de Madrid, 28049 Madrid, Spain
[6]Condensed Matter Physics Center (IFIMAC), Universidad Autónoma de Madrid, 28049 Madrid, Spain
[7]Laboratorio de Cristalografía, Departamento de Química, Facultad de Ciencias, Universidad de Los Andes, Mérida 5101, Venezuela
* Correspondence: ivan.brito@uantof.cl; Tel.: +56-55-2637814

Content	Page
**Table S1.** Zn(II) ion Bond Distances (Å) of compounds **1** and **2**.	2
**Table S2.** Zn(II) ion coordination sphere bond angles (º) of compounds **1** and **2**.	2
**Table S3.** Hydrogen bonds interactions of compounds **1**.	3
**Table S4.** Hydrogen bonds interactions of compounds **2**.	3
**Table S5.** Summary of the hydrogen bond distances (Å) and angles (°) for **2**.	4
**Figure S1.** Experimental powder diffractograms and Rietveld refinement plots of compounds **1** and **2**.	4
**Figure S2.** Experimental Powder diffractogram of compound **1** residue from TG experiment.	5

Table S1. Zn(II) ion Bond Distances (Å) of compound **1** and **2**.

**1**			**2**		
Atom	Atom	Length/Å	Atom	Atom	Length/Å
Zn1	O2	2.044(2)	Zn1	O2[1]	2.151(2)
Zn1	O2[1]	2.044(2)	Zn1	O2[2]	2.151(2)
Zn1	N1[2]	2.297(3)	Zn1	N1	2.142(3)
Zn1	N1[3]	2.297(3)	Zn1	N1[3]	2.142(3)
Zn1	N4[1]	2.151(3)	Zn1	N4[1]	2.166(3)
Zn1	N4	2.151(3)	Zn1	N4[2]	2.166(3)

[1]1/2-X,3/2-Y,-Z; [2]-1/2+X,3/2-Y,-1/2+Z; [3]1-X,+Y,1/2-Z.

Table S2. Zn(II) ion coordination sphere bond angles (º) of compounds **1** and **2**.

**1**				**2**			
Atom	Atom	Atom	Angle/˚	Atom	Atom	Atom	Angle/˚
O2	Zn1	O2[1]	180.00(11)	O2[1]	Zn1	O2[2]	174.64(12)
O2	Zn1	N1[2]	92.14(9)	O2[1]	Zn1	N4[2]	99.77(11)
O2	Zn1	N1[3]	87.86(9)	O2[1]	Zn1	N4[1]	76.32(10)
O2[1]	Zn1	N1[3]	92.14(9)	O2[2]	Zn1	N4[1]	76.32(10)
O2[1]	Zn1	N1[2]	87.86(9)	O2[2]	Zn1	N4[1]	99.77(11)
O2	Zn1	N4[1]	100.02(9)	N1[3]	Zn1	O2[1]	87.65(11)
O2[1]	Zn1	N4	100.02(9)	N1[3]	Zn1	O2[2]	96.16(11)
O2	Zn1	N4	79.98(9)	N1	Zn1	O2[2]	87.65(11)
O2[1]	Zn1	N4[1]	79.98(9)	N1	Zn1	O2[1]	96.16(11)
N1[2]	Zn1	N1[3]	180.0	N1[3]	Zn1	N1	89.57(17)
N4[1]	Zn1	N1[3]	83.00(9)	N1[3]	Zn1	N4[2]	93.10(12)
N4	Zn1	N1[2]	83.00(9)	N1[3]	Zn1	N4[1]	163.94(12)
N4[1]	Zn1	N1[2]	97.00(9)	N1	Zn1	N4[2]	163.94(11)
N4	Zn1	N1[3]	97.00(9)	N1	Zn1	N4[1]	93.10(12)
N4[1]	Zn1	N4	180.0	N4[1]	Zn1	N4[2]	88.70(16)

[1]+X,1+Y,+Z; [2]1-X,1+Y,1/2-Z; [3]1-X,+Y,1/2-Z; [4]+X,-1+Y,+Z.

Table S3. Hydrogen bonds interactions of compound **1**.

D	H	A	d(D-H)/Å	d(H-A)/Å	d(D-A)/Å	D-H-A/°
C3	H3	O2[1]	0.93	2.45	3.047(4)	122.4
C4	H4	O1[2]	0.93	2.48	3.163(4)	130.0
C6	H6A	O2[3]	0.97	2.67	3.391(4)	131.5
C10	H10A	N4[4]	0.96	2.78	3.424(4)	124.8
C10	H10B	O1	0.96	2.6	3.241(4)	124.5
C10	H10C	O1[5]	0.96	2.72	3.450(5)	132.9

[1]1/2+X,3/2-Y,1/2+Z; [2]1-X,1+Y,1/2-Z; [3]1/2+X,1/2+Y,+Z; [4]1-X,1-Y,-Z; [5]1-X,+Y,1/2-Z

Table S4. Hydrogen bonds interactions of compound **2**.

D	H	A	d(D-H)/Å	d(H-A)/Å	d(D-A)/Å	D-H-A/°
C4	H4	O2S[1]	0.93	2.87	3.463(7)	122.7
C5	H5	O1S[2]	0.93	2.84	3.455(6)	125.0
C6	H6A	N3[3]	0.97	2.43	3.343(5)	156.1
C10	H10B	O1	0.96	2.62	3.241(5)	122.9
O1S	H1SA	O1[4]	0.85	1.95	2.792(4)	173.2
O1S	H1SB	O1[5]	0.85	2.66	3.249(4)	127.9
O1S	H1SB	O2[5]	0.85	2.22	3.067(4)	171.5
O2S	H2SA	O1S	0.85	2.17	3.020(6)	173.7
O2S	H2SB	O1S[6]	0.85	2.13	2.979(6)	177.7

[1]1+X,1+Y,+Z; [2]3/2-X,3/2-Y,-Z; [3]1-X,1-Y,-Z; [4]+X,1-Y,-1/2+Z; [5]3/2-X,1/2+Y,1/2-Z; [6]3/2-X,1/2-Y,-Z

Table S5. Summary of the hydrogen bond distances (Å) and angles (°) for **2**.

D—H ···A	D – H	H···A	D – A	D – H ···A
O1S–H1SA···O1^{i}	0.85	1.95	2.792(4)	173.2
O1S–H1SB···O1^{ii}	0.85	2.66	3.249(4)	127.9
O1S–H1SB···O2^{ii}	0.85	2.22	3.067(4)	171.9
O1S–H1SA···O1S	0.85	2.17	3.020(4)	173.7
O1S–H1SA···O1S^{iii}	0.85	2.13	2.979(4)	177.7

(i) -x,-y+1,z-1/2, (ii) -x+3/2, y+1/2, -z+1/2, (iii) -x+3/2, -y+1/2, -z.

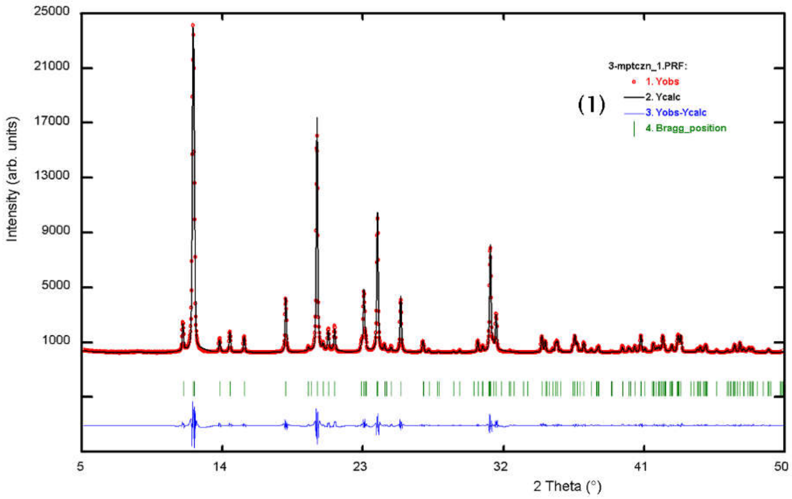

Rp= 6.6, Rwp= 8.7

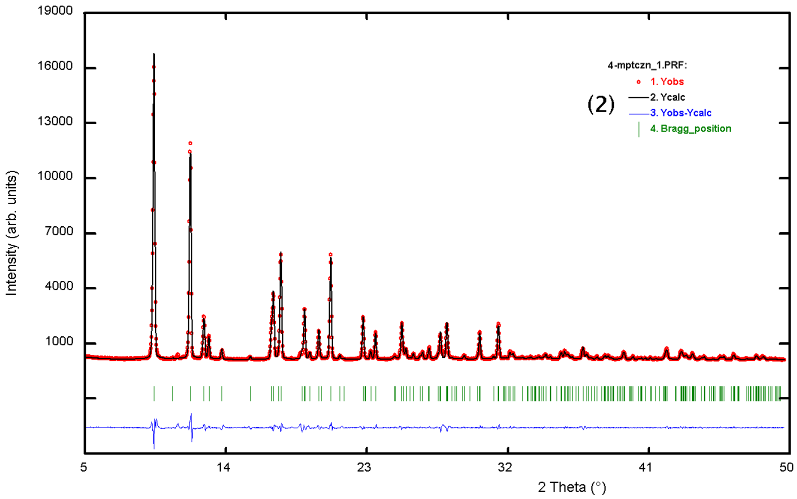

Rp= 5.9, Rwp= 7.8

Figure S1. X-ray powder diffraction patterns (**1**, **2**) and Rietveld refinement plots.

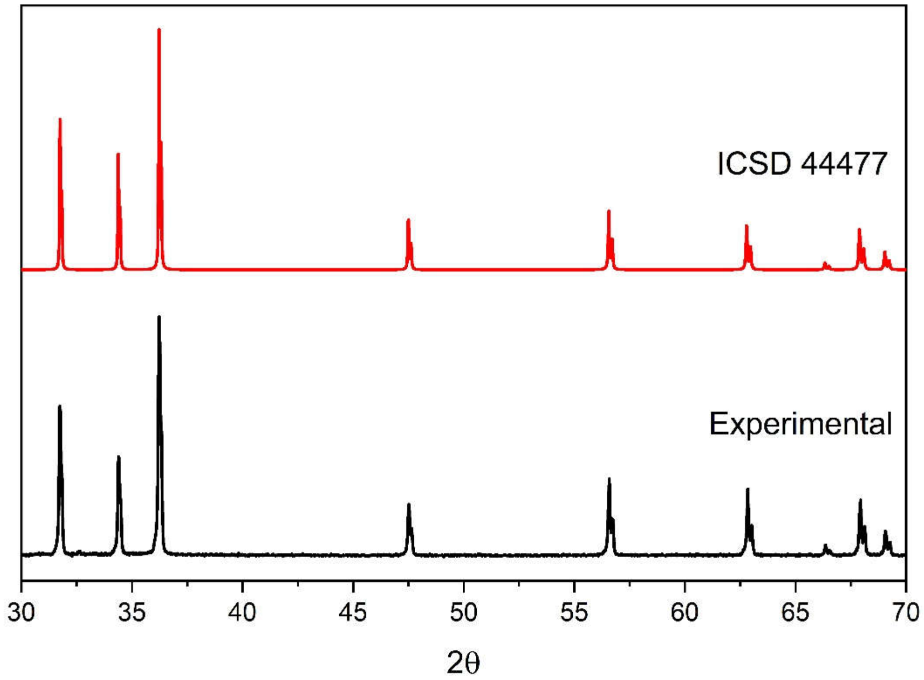

Figure S2. Experimental Powder diffractogram of compound **1** residue from TG experiment.
